# Sunbed Use Prevalence and Associated Skin Health Habits: Results of a Representative, Population-Based Survey among Austrian Residents

**DOI:** 10.3390/ijerph13020231

**Published:** 2016-02-19

**Authors:** Daniela Haluza, Stana Simic, Hanns Moshammer

**Affiliations:** 1 Institute of Environmental Health, Center for Public Health, Medical University of Vienna, Kinderspitalgasse 15, Vienna A-1090, Austria; hanns.moshammer@meduniwien.ac.at; 2 Institute of Meteorology, University of Natural Resources and Life Sciences, Vienna, Peter-Jordan-Straße 82, Vienna A-1190, Austria; stana.simic@boku.ac.at

**Keywords:** public health, preventive medicine, sun exposure, photo-protection, gender

## Abstract

Recreational sunbed use accounts for the main non-solar source of exposure to ultraviolet radiation in fair-skinned Western populations. Indoor tanning is associated with increased risks for acute and chronic dermatological diseases. The current community-based study assessed the one-year prevalence of sunbed use and associated skin health habits among a representative, gender-balanced sample of 1500 Austrian citizens. Overall one-year prevalence of sunbed use was 8.9% (95% confidence interval (CI) 7.5%–10.4%), with slightly higher prevalence in females (9.2%, 95% CI 7.3%–11.2%) compared to males (8.6%, 95% CI 6.7%–10.6%). Factors predicting sunbed use were younger age (by trend decreasing with older age), place of living, smoking, skin type (by trend increasing with darker skin), sun exposure, motives to tan, and use of UV-free tanning products. Despite media campaigns on the harmful effects of excessive sunlight and sunbed exposure, we found a high prevalence of self-reported sunbed use among Austrian citizens. From a Public (Skin) Health perspective, the current research extends the understanding of prevailing leisure time skin health habits in adding data on prevalence of sunbed use in the general Austrian population.

## 1. Introduction

From an evolutionary perspective, fair skin is seen as a selection advantage in regions with low solar intensity by preventing vitamin D deficiency, thus, increasing longevity and fertility as well as preventing autoimmune diseases and cancer development [1,2]. Exposure to natural and artificial ultraviolet radiation (UVR) initiates the physiological process of skin tanning, which is associated with epidermal cells damage [3]. A growing body of scientific evidence argues for the carcinogenic properties of indoor exposure to ultraviolet radiation [4,5,6,7,8,9]. However, the popularity of indoor tanning as recreational pastimes is hardly on the wane in Western countries with mainly fair-skinned populations [10,11,12]. Wehner *et al.* estimated that over 400,000 non-melanoma and 10,000 melanoma cases each year are attributable to sunbed use in the U.S., Europe, and Australia [13].

Worldwide, several countries implemented more stringent policies and programs to mitigate sunbed use, increase customer stewardship, raise awareness, and provoke action by government and industry [14,15]. Recently, the number of countries restricting underage youth access to indoor tanning facilities increased from two countries (Brazil, France) in 2003 to eleven countries in 2011, now including Austria, Belgium, Germany, Portugal, Spain, and the U.K. in Europe, and parts of Canada, the U.S, and Australia [15].

Limiting UVR exposure and increasing sun protection could reduce skin cancer incidence rates and associated healthcare costs [16,17]. Awareness campaigns as an important tool for skin cancer prevention influence tanning attitudes and educate the public about appropriate photo-protection [18,19]. The effect of these campaigns in diminishing recreational sunbed use, potentially due to vast media and public policy attention, seems encouraging [20,21].

Based on the broader concept of Public Health, the umbrella term Public (Skin) Health refers to skin health-related activities aimed at lowering incidence rates of photo-induced skin manifestations [22,23,24,25,26,27]. By encouraging individuals to adopt skin health-sensitive behaviors, Public (Skin) Health has practical implications for community-based skin health promotion. Focusing on potential risks accompanied with recreational exposure to solar and artificial UVR, the respective research activities define risk groups as target for according educative campaigns and public policies. In this context, Bock *et al.* identified a need for studies examining behavioral patterns related to sunbed use on a national level to develop successful skin health promotion strategies [21].

Similar to other countries, Austrian melanoma incidence rates (1983: 4.8; 2012: 12.3/100,000) and mortality rates (1983: 1.9, 2012: 2.2/100,000) have constantly increased over the last decades [28,29]. Despite these rising skin cancer rates, little is known about the national prevalence of sunbed use and the associated skin health burden in Austria as a potential lifestyle factor influencing the individual skin cancer risk. To close this knowledge gap, we conducted the population-based UV Skin Risk survey to assess prevailing intentional tanning behaviors including sunbed exposure as well as sunless tanning product use. Thus, this article specifically analyzed socio-demographic characteristics, knowledge, and attitudes of sunbed users in comparison to non-users.

## 2. Methods

### 2.1. Study Population

The current study presents data on prevailing indoor and outdoor tanning practices in relation to skin health knowledge, attitudes, and motives collected by the cross-sectional, population-based UVSkinRisk survey. This study was performed in accordance with the ethical standards of the Declaration of Helsinki and was approved by the local ethics committee of the Medical University of Vienna. Further methodological details are given elsewhere [25,26,27,29,30]. In short, we contracted the independent, Vienna-based market research company Triconsult to conduct the questionnaire-based telephone survey in August 2011. As a third party, Triconsult also ensured quality control and respondents’ anonymity. The study sample represented the target population of the German-speaking general Austrian population according to the national 2011 census data regarding age, population of federal states, and population size of place of residence [31,32]. We used stratified random sampling on the basis of 20,000 telephone numbers from the official national telephone directory list. About 11,100 individuals were contacted to finally reach the pre-set number of 1500 Austrian male and female survey participants representing the general population. Verbal consent was obtained from all respondents prior to the telephone interview. Participation in the questionnaire-based telephone interview of approximately 10–12 min in length was voluntarily and anonymous.

### 2.2. Survey Questionnaire

The questionnaire collected socio-demographic data including age, gender, place of residence, city size, highest attained education, and occupational situation as well as personal status. All Austrian geographic regions were represented in the data set and were assigned to the geographic regions of Austria, *i.e.*, East, South, and West [33]. As suggested by Grange *et al.*, we allocated type of occupation to three socio-professional categories (SPC), namely SPC+ (white-collar and freelance work), SPC− (manual work and domestic service), and retired/unemployed [11]. We categorized the highest attained education level as primary, secondary, and tertiary education.

We also collected self-reported information on general sunbed use (no/yes) by the question “In general, do you use sunbeds?” We assigned study participants who declared general sunbed use to the group of “recent sunbed users”. The remaining participants were considered as “no recent sunbed users”, as suggested by the literature [34]. The study questionnaire also assessed frequency of seasonal sunbed use, *i.e.*, in winter (October to March) and summer (April to September). Given that fake tanning options providing a tanned appearance without UVR exposure are very popular, we asked for prevalent use of these sunless tanning products [35,36]. The according multiple-answer question “Do you usually use UV-free tanning products?” offered the possible choices self-tanning/bronzer lotions (e.g., dihydroxyacetone-based products), nutritional supplements (e.g., carotenoids), temporary bronzers, tanning accelerators/tan enhancers (e.g., tyrosine-based products), and also medical products (e.g. melanotan peptide hormones).

Participants classified their skin type (phototype I-VI) according to the Fitzpatrick scale [37]. We assessed occurrence of sunburn in the past year as well as personal and family skin cancer history (all: no/yes) [38]. Participants provided information on frequency of sun protection use from always (=1) to never (=5) by a five-point Likert scale. In addition, respondents rated their degree of agreement with motives to tan using a five-point Likert scale ranging from strongly agree (=1) to strongly disagree (= 5). We summed the set of scores and calculated the mean of the respective items for generating the covariates sun protection and motives to tan. These covariates showed an internal consistency of Cronbach’s alpha = 0.64 and 0.73, respectively, which we considered to be acceptable.

To assess sources of skin health information, participants were asked to indicate their familiarity with skin health information material published by healthcare providers, indoor tanning studios, and sunscreen producers [22,30]. A test of true-false questions measured participants’ skin health knowledge. Summed amount of correct responses built the covariate knowledge score with higher scores indicating better knowledge.

We were further interested in self-reported lifestyle habits. We assessed information on current smoking status (non-smoking, ex-smoking, and smoking). Also, we collected data on regular outdoor sport activity (no/yes) and connectedness to nature ranging from none (=0) to very high (=10) as a measure for spending times outside and thus, recreational outdoor sun exposure [25]. We assessed sunbathing frequency and assigned participants to one of the two groups “no sun exposure” (0–5 days of sun exposure) and “sun exposure” (>5 days of sun exposure) [27]. We also asked for information on duration of sun exposure (ranging from <30 min to >3 h) and duration of sun exposure during midday hours (spanning from <30 min to >4 h).

### 2.3. Statistical Analysis

Median splitting dichotomized variables to create low and high connectedness to nature, skin health knowledge, motives to tan, and sun protection. For the latter two variables, low/high specifications were interpreted as more/less, respectively [25]. To compute prevalence estimates for sunbed use, we used the bootstrapping method based on 1000 bootstrap samples. We analyzed proportions, means, and standard deviation (SD) values of socio-demographic factors, skin health characteristics, attitudes, knowledge, and habits.

Group comparisons by each factor were done using chi²-tests, and two-sided level of significance was set at α = 0.05. Variables that statistically significantly differ in these group comparisons were further included as possible predictor variables for sunbed use in binary logistic regression analyses. We performed stepwise regression to keep the most relevant model according the AIC criterion. We conducted both crude and adjusted regression analysis and only reported odds ratio (OR) and 95% confidence intervals (95% CI) of adjusted results. Nagelkerke’s R^2^ and Hosmer-Lemeshow goodness-of-fit tests examined how well the regression model fitted the data set. All statistical analyses were performed using SPSS Version 21.0 (SPSS Inc., Chicago, IL, USA).

## 3. Results

In total, 1500 study subjects (50.5% females, 18–74 years, mean 44.7, SD 15.4 years) participated in this questionnaire survey. Table 1 presents the overall distribution of seasonal prevalence of sunbed use during winter and summer months. As expected, sunbed use was statistically significantly more common and frequent during winter (*p* < 0.001).

Table 2 shows basic characteristics of the study population in relation to sunbed use. Overall one-year prevalence of sunbed use was 8.9% (95% CI 7.5%–10.4%). We found a slightly higher prevalence in females (9.2%, 95% CI 7.3%–11.2%) compared to males (8.6%, 95% CI 6.7%–10.6%), but this gender difference did not reach statistical significance. Prevalences were highest in the youngest age group (18–29 years, 13.4% (95% CI 9.5%–17.7%), with a statistically significant linear decrease with age: 30–39 years: 11.9% (7.9%–15.8%), 40–49 years: 9.1% (6.2%–12.4%), 50-59 years: 7.3% (4.2%–10.8%), and 60–74 years: 3.2% (1.3%–5.4%), respectively (*p* < 0.001). Also, participants living in the East of Austria were more likely to be among the group of sunbed users (*p* < 0.045).

Table 3 shows prevalent sunbed use according to individual skin health characteristics and habits. Compared to non-users, sunbed users were more likely to smoke, have darker skin pigmentation (skin type III+), low connectedness to nature (all *p* < 0.05), outdoor sun exposure, more motives to tan, and also use of UV-free tanning products (all *p* < 0.001).

Item-based specifications showed that besides more consumption of UV-free tanning products in general, sunbed users also reported more use of temporary bronzers and tanning accelerators/tan enhancers (Table 4). However, non-recent sunbed users were more likely to consume nutritional supplements as endogenous tanning agents (*p* < 0.04). Furthermore, skin health information by healthcare providers was more often gathered among non-recent sunbed users (*p* < 0.012), whereas indoor tanning studios as information source was more common among users (*p* < 0.001). Knowledge on risk of sunbed use was higher among non-users (*p* < 0.001), while users were more aware of skin aging due to sun exposure (*p* < 0.028). Agreements with statements regarding motives to tan were consistently higher in the sunbed user group (all *p* < 0.05), except for concerns about acne (*p* = n.s.). Regarding sun protection habits, sunbed users were less likely to seek shade and wear a hat or sun-protective garments (all *p* < 0.05).

Table 5 depicts the results of the adjusted binary regression model (Hosmer-Lemeshow-Test: chi² = 2.965, *p* = 0.937). Nagelkerke’s R² suggested that the model explains 13% of the variation in the outcome and that the model was a good fit to the data (*p* > 0.05). The overall model predictive ability was about 91%. Factors predicting sunbed use were age, by trend decreasing with older age (for 60–74 year olds: OR = 0.30, 95% CI 0.14–0.62, *p* < 0.001), place of living by geographic regions of Austria (East *vs.* South, OR = 1.58, 95% CI 1.04–2.41, *p* < 0.032), smoking (non-smoking *vs.* current smoking, OR = 1.87, 95% CI 1.22–2.87, *p* < 0.004), skin type (skin type I *vs.* darker skin, by trend increasing with darker skin, *p* < 0.026), more sun exposure (OR = 1.69, 95% CI 1.13–2.51, *p* < 0.01), more motives to tan (OR = 2.10, 95% CI 1.40–3.15, *p* < 0.001), use of UV-free tanning products (OR = 1.82, 95% CI 1.11–2.98, *p* < 0.018).

## 4. Discussion

So far, little is known on potential lifestyle-associated explanations for rising skin cancer incidence and mortality rates in Austria. A considerable amount of studies have investigated prevalence of sunbed use, characteristics, attitudes, and knowledge of sunbed users in other European countries [8,11,21,34,39]. To close this knowledge gap, the present population-based study assessed prevalent general and seasonal sunbed use as well as skin health knowledge, attitudes, and habits among Austrian citizens. The practical and theoretical implications of these findings are discussed from a Public (Skin) Health perspective.

In a recently published meta-analysis including U.S., European, and Australian studies, prevalence of past-year sunbed use was 14% for adults, 18% for adolescents, and as high as 43% for university students [13]. In our sample, past-year sunbed use prevalence was 9% in general and 13% among the youngest age group (18–29 years). However, varying prevalence rates of ever sunbed use were reported across Europe. Schneider *et al.* identified as many as 47% ever and 21% current users among German adults [40]. These rates were higher compared to French data, where 13% of the general population reported having tanned indoors at least once in their lifetime and 4% in the past year, with higher rates (10%) among the younger population (20–25 years) [39].

To assess predictors of sunbed use, we employed an adjusted regression model showing both an adequate goodness-of-fit to the data and strong predictive power. Sunbed users were typically younger, lived in the Eastern region of Austria, smoked cigarettes, used UV-free tanning products, and reported darker skin, outdoor sun exposure, and more motives to tan. These socio-economic profiles of recent sunbed users have not been addressed in national skin health awareness campaigns in Austria so far. Approaches sensitive to recipients’ socio-economic background and educational level might more successfully increase general skin cancer risk awareness and also reduce sunbed use.

Sunbed exposure is a risk factor for melanoma even among persons who never experienced sunburns from indoor or outdoor UVR contact [41]. Although a tan does not protect against sunburn, individuals often tan indoors before planned sun exposure, presumably to prevent sunburns [10]. Alternatively, a wide range of UV-free tanning products are commercially available and commonly used to boost the tanning process. Their consumption is associated with more frequent use and higher risk for tanning addiction [36].

We found a respective prevalence of about 11% and statistically significant higher use of UV-free tanning products among sunbed users compared to non-users. Also, their self-reported consumption predicted sunbed use in our regression model. To our knowledge, this is the first study evaluating the prevailing use of these products among Austrian citizens. This finding is of relevance for skin health counseling and could be further integrated in future implementation of labeling requirements for these products, e.g., showing skin health messages. Day *et al.* differentiated between three distinct types of tanning behavior: outdoor tanners, fake tanners, and tan avoiders [35]. This differentiation was beyond the scope of our study, as we aimed at providing so far lacking baseline data on recreational skin health behavior among the Austrian population. However, evaluating personal tanning preferences and their interaction with long-term skin health outcomes may reveal novel Public (Skin) Health strategies.

Recently, we reported on geographic differences in melanoma incidence and mortality trends in Austria [29]. In addition to this, the current analysis revealed that prevalence of sunbed use among Austria citizens followed an East-West gradient. This regional gradient has already been shown for various lifestyle-associated health determinants such as cardiovascular diseases, diabetes mellitus, and obesity [42]. Urban-rural influences on socio-economic status and recreational habits might explain this well-established association. However, a South-North gradient with higher prevalence of sunbed exposure in the North compared to the South was found in several European countries [34,43]. This so far unknown result on geographical associations with sun exposure habits in Austria could motivate future hypothesis-driven evaluations to identify novel strategies and risk groups for targeted awareness campaigns.

Ezzedine *et al.* found a correlation between indoor and outdoor UVR exposure habits and lifestyle habits [34]. In agreement with Grange *et al.*, our analysis showed that sunbed users were less likely to use sun-protective measures such as hats or clothes than non-users [11]. Further, we observed higher sunbed use prevalence in younger study subjects. Accordingly, several publications identified the typical sunbed user as being female, of younger age and—beyond this—having a higher socio-professional category and a fairer phototype [11,34,40,43,44]. Nevertheless, Schneider *et al.* found higher prevalence rates among individuals with medium education, whereas age, partnership status, and nationality were not associated with sunbed use [40]. Exposure to artificial UVR increases the risk of skin cancer, irrespective of age of initial indoor tanning [7,45]. Boniol and colleagues reported that melanoma risk was higher if first exposure to indoor tanning equipment occurred before the age of 35 years [8]. Initiation of sunbed use at young adult age suggests the need for targeted interventions. In particular, adolescents should be made aware of the long-term skin health risks of sunbed use when used for short-term cosmetic tanning purposes [15].

Our analysis also found that sunbed use was associated with smoking habits and outdoor sun exposure. This is in line with vast scientific evidence, showing that sunbed use is correlated with risky lifestyle habits including smoking cigarettes, drinking alcohol, eating unhealthy food, and sunbathing and thus, accumulating risk factors for skin health [11,34,40,44,46]. In this context, Gunn *et al.* verified that smoking and sunbed use are strongly associated with photoaging and wrinkling in both genders, while a reasonable lifestyle preserves youthful looks on the long run [47].

Appearance- and emotion-based motives to tan influence both solar and indoor tanning habits [48]. Although sunbed users were shown to know that sun exposure reduces the skin’s regenerative capacity, they consider that a tan makes a person look more attractive and protects the skin [11,34]. In our survey, sunbed users compared to non-users were more aware of the risk of photo-induced skin aging, but they perceived a lower skin health risk in connection with sunbed use. Likewise, agreements with statements regarding motives to tan were consistently higher in the sunbed user group. This observation suggests that research entangling motives of indoor tanners could provide valuable input for larger-scale skin cancer prevention policies and monitoring programs [7].

Knowledge, attitudes, and intentions of individuals are vital targets for public education programs. However, there is still a lack of information among consumers regarding the safety of sunbeds use [46]. Schneider *et al.* reported alarmingly poor quality of services provided by tanning parlors [40]. In our study, non-recent sunbed users more often received skin health information by healthcare providers, whereas users more often mentioned indoor tanning studios as a source of information. Given the known publishing source bias of information material, these finding suggests the need for standardizing the content of skin health educative information [23].

Sunbed users experience positive emotions and relaxation during frequent and intentional exposure to artificial UVR, potentially leading to tanning addiction, also referred to as tanorexia [49]. This concept of tanorexia is supported by the observation that the annual exposure remained constant over time among German sunbed users [21]. Contrarily, Guy *et al.* reported a recent decrease in sunbed use among U.S. adults [20]. This decline might reflect increased public awareness of the carcinogenic potential sunbed use due to respective skin health campaigns and implementation of laws restricting sunbed access among minors [14,16]. Characteristics associated with sunbed use cessation include greater awareness of skin cancer risk and higher educational level [21]. Evidence suggests that positive attitudes towards tanning are a key barrier to adopt measures towards preventing skin health hazards [21,48]. In addition to tackling these pro-tanning societal framing and motives to tan, awareness campaigns focusing on sunbed use cessation should also account for gender—and age—specific requirements.

Besides the aforementioned Public (Skin) Health aspects of sunbed use, there is evidence of detrimental risks of UVR exposure avoidance contributing to a higher risk for all-cause mortality [50,51]. These photo-induced benefits are probably due to the still controversially discussed Vitamin D-associated health effects [52,53]. Nevertheless, according to Woo *et al.*, Vitamin D supplementation is a feasible means for adequate Vitamin D supply while avoiding sunbed exposure as a the risk factor for skin cancer and skin aging [54].

The present survey is the first empirical study that collected data from a large, community-based sample representing the Austrian census data [26]. Amount of participants (*n* = 1500) was equal to comparable European studies such as the French EDIFICE Melanoma survey (*n* = 1502), although France has a far larger population size [11,55]. Public (Skin) Health research has wide implications for clinical practice and community skin health promotion. Closing the knowledge gap regarding sunbed use in Austria, the current study theoretically advances the understanding of prevailing skin health habits in the general Austrian population.

However, the findings of this study are subject to several limitations, mainly related to the cross-sectional design. Thus, the survey data do not allow for causal relations of individual characteristics and indoor tanning behaviors. We used stratified random sampling on the basis of the official national telephone directory list to ensure representativeness of the study population. However, as in every telephone-based survey, potential study participants needed to have a valid telephone number when contacted, thus introducing selection bias and limiting generalizability of the study results to the general adult population. Also, we assessed self-reported data, which might be subject to non-response, reporting, and recall bias. Nevertheless, recall bias regarding UVR exposure seems to be small and self-reported data on phenotypic characteristics, sunburn history, and sun protection behavior were shown to be reproducible [56,57]. We assume that these data represent a trustworthy picture of actual skin health habits executed by the Austrian population. Although our data on recent sunbed users were based on the respective group of only about 9% of study participants, they were comparable with sunbed use prevalence reported in other studies, e.g., Grange *et al.* [11]. Thus, we also used the bootstrapping method based on 1000 bootstrap samples to additionally provide prevalence estimates for sunbed use in Austria. The herein presented data may serve as a baseline for tracking progress achieved by future Public (Skin) Health campaigns, as suggested by Davis *et al.* [58]. Since September 2010, Austria has implemented a legislation to ban indoor tanning bed use for minors nationwide [59]. Further research could analyze the impact of this ban on skin health-related longitudinal trends and behavior changes in the general population.

## 5. Conclusions

The current research extends the understanding of prevailing recreational skin health habits by adding hitherto lacking data on sunbed use prevalence in the general Austrian population. Sunbed users were typically young, smoke cigarettes, use UV-free tanning products, and sunbathe outdoors. Also, prevalence of sunbed use in Austria followed an East-West gradient. National skin health awareness campaigns have not yet address these at-risk profiles for sunbed use. From a Public (Skin) Health perspective, approaches sensitive to recipients` socio-economic background and educational level might more successfully increase general skin cancer risk awareness and also reduce sunbed use.

## Figures and Tables

**Table 1:** Seasonal one-year prevalence of sunbed use.

Sunbed use	Season *			
	Winter		Summer	
	N	%	N	%
Overall	1500	100.0	1500	100.0
No recent sunbed use	1366	91.1	1366	91.1
Recent sunbed use	134	8.9	134	8.9
No seasonal sunbed use	12	0.8	69	4.6
Once a month	45	3.0	23	1.5
More than once a month	45	3.0	27	1.8
Once a week	27	1.8	12	0.8
More than once a week	5	0.3	3	0.2

Notes: * Winter: October–March, summer: April–September.

**Table 2:** Socio-demographic characteristics of the study population, stratified by sunbed use.

Factor	N	No Recent Sunbed Use		Recent Sunbed Use		*p*-Value (chi²)
		N	%	N	%	
Gender						
Female	758	688	90.8	70	9.2	
Male	742	678	91.4	64	8.6	0.679
Age (years)						
18–29	305	264	86.6	41	13.4	
30–39	278	245	88.1	33	11.9	
40–49	340	309	90.9	31	9.1	
50–59	260	241	92.7	19	7.3	
60–74	317	307	96.8	10	3.2	0.001 **
Geographic region in Austria						
East	644	573	89.0	71	11.0	
South	320	298	93.1	22	6.9	
West	536	495	92.4	41	7.6	0.045 *
Place of residence						
Rural	1020	927	90.9	93	9.1	
Urban	480	439	91.5	41	8.5	0.715
Education level						
Primary	357	329	92.2	28	7.8	
Secondary	706	634	89.8	72	10.2	
Tertiary	437	403	92.2	34	7.8	0.269
Socio-professional category (SPC)						
SPC+	572	517	90.4	55	9.6	
SPC−	475	429	90.3	46	9.7	
Retired/unemployed	453	420	92.7	33	7.3	0.338
Personal status						
Single	507	456	89.9	51	10.1	
Partner	993	910	91.6	83	8.4	0.275

Notes: * *p* < 0.05; ** *p* < 0.001.

**Table 3:** Health behavior and skin health characteristics of the study samples, stratified by sunbed use.

Factor	N	No Recent Sunbed Use		Recent Sunbed Use		*p*-Value (chi²)
		N	%	N	%	
Smoking						
Non-smoking	841	782	93.0	59	7.0	
Ex-smoking	313	285	91.1	28	8.9	
Smoking	346	299	86.4	47	13.6	0.002 *
Skin type						
I	79	76	96.2	3	3.8	
II	441	411	93.2	30	6.8	
III	657	585	89.0	72	11.0	
IV–VI	323	294	91.0	29	9.0	0.040 *
Skin cancer history						
Personal						
Yes	230	215	93.5	15	6.5	
No	1270	1151	90.6	119	9.4	0.163
Family						
Yes	147	138	93.9	9	6.1	
No	1353	1228	90.8	125	9.2	0.208
Sunburn						
Yes	461	410	88.9	51	11.1	
No	1039	956	92.0	83	8.0	0.054
Sun exposure						
Yes	707	619	87.6	88	12.4	
No	793	747	94.2	46	5.8	0.001 **
Sun protection						
Low	722	666	92.2	56	7.8	
High	778	700	90.0	78	10.0	0.124
Motives to tan						
Low	732	638	87.2	94	12.8	
High	768	728	94.8	40	5.2	0.001 **
Connectedness to nature						
Low	475	418	88.0	57	12.0	
High	1025	948	92.5	77	7.5	0.005 *
Skin health knowledge						
Low	319	290	90.9	29	9.1	
High	1181	1076	91.1	105	8.9	0.911
Sport activity						
Yes	942	848	90.0	94	10.0	
No	558	518	92.8	40	7.2	0.065
UV-free tanning						
Yes	159	134	84.3	25	15.7	
No	1341	1232	91.9	109	8.1	0.001 **

Notes: * *p* < 0.05; ** *p* < 0.001.

**Table 4:** Distribution of tanning attitudes, skin health knowledge, and sun protective habits, stratified by sunbed use.

Factor	Total	No Recent Sunbed Use		Recent Sunbed Use		*p*-Value (chi²)
	N	N	%	N	%	
UV-free tanning products						
No use	1341	1232	90.2	109	81.3	0.001 **
Self-tanning/bronzer lotions	108	93	6.8	15	11.2	0.061
Nutritional supplements	31	93	6.8	6	4.5	0.040 *
Temporary bronzers	24	25	1.8	6	4.5	0.005 *
Tanning accelerator/tan enhancers	13	18	1.3	5	3.7	0.001 **
Medical products	4	8	0.6	1	0.7	0.259
Skin health information publisher						
Healthcare providers	1247	1146	83.9	101	75.4	0.012 *
Indoor tanning studios	160	96	7.0	64	47.8	0.001 **
Sunscreen producers	804	725	53.1	79	59.0	0.193
Skin health knowledge						
Risks of sunbed use	1190	1105	80.9	85	63.4	0.001 **
Risks of sun exposure	1187	1088	79.6	99	73.9	0.117
Skin aging risk of sun exposure	1281	1158	84.8	123	91.8	0.028 *
Sunburn risk for tanned skin	1260	1141	83.5	119	88.8	0.112
Motives to tan: When I am tanned…						
I have more sex appeal.	1069	955	69.9	114	85.1	0.001 **
I am more attractive.	679	584	42.8	95	70.9	0.001 **
I am more self-confident.	405	347	25.4	58	43.3	0.001 **
I am not so pale.	796	690	50.5	106	79.1	0.001 **
I am less concerned about acne.	333	296	21.7	37	27.6	0.114
I am less concerned about stretch marks.	248	214	15.7	34	25.4	0.004 *
I look slimmer.	644	572	41.9	72	53.7	0.008 *
I look fitter.	298	262	19.2	36	26.9	0.033 *
Sun protection: For sun protection…						
I use sunscreen (min. SPF 15).	996	907	66.4	89	66.4	0.996
I seek shade.	1219	1129	82.7	90	67.2	0.001 **
I wear a hat.	732	681	49.9	51	38.1	0.009 *
I wear sunglasses.	1136	1033	75.6	103	76.9	0.749
I wear sun-protective garments.	793	737	54.0	56	41.8	0.007 *
I avoid midday sun.	878	810	59.3	68	50.7	0.055

Notes: * *p* < 0.05; ** *p* < 0.001.

**Table 5:** Results of the linear regression analysis on sunbed use.

Factor	OR	95% CI	*p*-Value
Age			
18–29	1	-	0.014 *
30–39	1.02	0.61–1.72	0.934
40–49	0.84	0.49–1.41	0.504
50–59	0.68	0.37–1.24	0.207
60–74	0.30	0.14–0.62	0.001 **
Geographic regions of Austria			
East	1	-	0.040 *
South	1.58	1.04–2.41	0.032 *
West	0.95	0.54–1.65	0.845
Smoking			
Non-smoking	1	-	0.014 *
Ex-smoking	1.44	0.88–2.35	0.149
Smoking	1.87	1.22–2.87	0.004 *
Skin type			
I	1	-	0.026 *
II	1.35	0.39–4.63	0.635
III	2.58	0.78–8.57	0.123
IV-VI	1.89	0.54–6.55	0.317
Sun exposure	1.69	1.13–2.51	0.010 *
Motives to tan	2.10	1.40–3.15	0.001 **
Connectedness to nature	1.39	0.94–2.03	0.096
UV-free tanning	1.82	1.11–2.98	0.018 *

Notes: * *p* < 0.05; ** *p* < 0.001.
